# Prevalence and associated risk factors of hypertension amongst adults in a rural community of Limpopo Province, South Africa

**DOI:** 10.4102/phcfm.v7i1.847

**Published:** 2015-10-22

**Authors:** Sam T. Ntuli, Eric Maimela, Mariannes Alberts, Solly Choma, Sekgothe Dikotope

**Affiliations:** 1 Research Development and Administration, University of Limpopo, Polokwane Campus, South Africa; 2 Department of Public Health, University of Limpopo, Polokwane Campus, South Africa; 3 Epidemiology Services, Limpopo Department of Health, South Africa; 4 Medical Science Department, University of Limpopo, Turfloop Campus, South Africa

## Abstract

**Background:**

Hypertension is problem already faced by urban populations of South Africa, but little is known about its prevalence and risk factors in rural areas.

**Aim:**

To assess the prevalence of and risk factors associated with hypertension amongst adults in a rural community in South Africa.

**Setting:**

Dikgale Health and Demographic Surveillance Site, Limpopo Province, South Africa.

**Methods:**

A community-based cross-sectional survey was carried out at this site where individuals aged 15 years and older were screened using a locally adapted version of the World Health Organization STEPwise questionnaire. Demographics, anthropometry and three independent blood pressure (BP) readings were taken. The average of the three BP measurements was used in analysis, and hypertension taken as systolic and diastolic BP of ≥ 140 or ≥ 90 mmHg respectively, or at least a two-week history of antihypertensive treatment. Analysis included the Chi-square test and statistical significance was set at *p* ≤ 0.05.

**Results:**

A total of 1407 individuals were interviewed, of whom 1281 had complete BP, weight and height measurements taken. The mean age of participants was 44.2 ± 20.9 years(range 15–98 years), 63% were female, 55% were single and 90% were unemployed, whilst 13% were tobacco smokers and 20% reported drinking alcohol. Overall prevalence of hypertension was 41% and this was significantly associated with age and marital status.

**Conclusion:**

The prevalence of hypertension was found to be high. Prevention strategies are urgently needed to address this life-threatening and important risk factor for cardiovascular disease in rural Limpopo Province.

## Abstrak

**Prévalence de l’hypertension et facteurs de risque associés chez les adultes dans une communauté rurale de la province du Limpopo en Afrique du Sud.**

**Objectif:**

Évaluer la prévalence et les facteurs de risque associés à l’hypertension chez les adultes au Site de surveillance sanitaire et démographique de Dikgale, dans la province du Limpopo.

**Méthodes:**

Une enquête transversale à base communautaire a été réalisée au Site de surveillance sanitaire et démographique de Dikgale, dans la province du Limpopo en Afrique du Sud. Des personnes âgées de 15 ans et plus ont été sélectionnées en utilisant une version adaptée localement du questionnaire STEPwise de l’OMS. Des relevés démographiques, anthropométriques et trois relevés indépendants de pression artérielle (PA) ont été effectués lors d’une visite au domicile de chaque participant. La moyenne des trois mesures de PA été utilisée dans l’analyse. L’hypertension a été définie selon des critères du JNC 7 : pressions artérielles systolique et diastolique ≥ 140 ou ≥ 90 mmHg respectivement, ou lorsqu’un participant a eu au moins un antécédent de deux semaines de traitement antihypertenseur. L’analyse comprenait l’utilisation du test du chi deux et la signification statistique a été fixée à p ≤ 0,05.

**Résultats:**

Un total de 1407 personnes ont été interrogées, dont 1281 ont fait l’objet de mesures complètes de PA, du poids et de la taille qui ont été analysées. L’âge moyen des participants était de 44.2 ± 20.9 ans (éventail de 15 à 98 ans), 63% étaient des femmes, 55% étaient célibataires et 90% étaient au chômage. En ce qui concerne les habitudes alimentaires des participants, 89% mangeaient moins de cinq portions de fruits et légumes par jour, 13% fumaient du tabac et 20% déclaraient boire de l’alcool. La prévalence globale de l’hypertension était de 41% dans l’échantillon des participants et était associée de façon significative à l’âge et au statut matrimonial. Le sexe, la consommation d’alcool, le tabagisme, la situation professionnelle, la consommation de fruits et légumes et l’Indice de masse corporelle (IMC) n’étaient pas liés à une hypertension. La moitié des participants avaient un IMC de 25 kg/m2 ou plus et parmi ceux-ci, 27% étaient en surpoids (IMC de 25.0 – 29.9) et 23% étaient obèses (IMC ≥ 30 kg/m2).

**Conclusion:**

La prévalence de l’hypertension a été jugée élevée dans cette étude. Des stratégies de prévention doivent urgemment être mises en œuvre pour remédier à ce facteur de risque important et potentiellement mortel en termes de maladies cardio-vasculaires dans la province rurale du Limpopo en Afrique du Sud.

## Introduction

Cardiovascular disease (CVD) is now the leading cause of death worldwide, and its major impact is not only observed in developed countries but also in developing countries.^1,2^ In 2008 CVD caused 30% of all global deaths, of which over 80% were recorded from developing countries.^3^ Many developing countries are now in a phase of epidemiological transition and face the double burden of infectious diseases and non-communicable diseases (NCDs). Bradshaw and colleagues reported that hypertension, strokes and ischaemic heart disease are amongst the most common NCDs causing many premature adult deaths in South Africa.^4^ Various studies have identified CVD as a major burden of disease in developing countries.^5,6,7^

In recent years epidemiological evidence has increasingly provided more insight into the risk factors for CVD in developing countries. These include, amongst others, obesity, alcohol intake, smoking, hypertension and diabetes mellitus.^1,8,9^ Hypertension remains the most common life-threatening risk factor for CVD in developing countries.^10^ This has major cost implications for low- and middle-income countries and requires urgent strategies for prevention and management of the disease. Numerous studies have been conducted worldwide to estimate the prevalence of hypertension. In sub-Saharan countries the prevalence of hypertension varies from 5% to 50%,^11,12^ whilst in economically developed countries it ranges between 19% and 30%.^4,13^

In South Africa the prevalence of hypertension was estimated at 21% in the 1998 Demographic and Health Survey, and it was slightly more common in women (14%) than in men (11%).^14^ Newer studies suggest a much higher prevalence of hypertension, in line with the epidemiological transition and increasing burden of chronic diseases of lifestyle in South Africa.^15,16,17,18^ Despite the importance of hypertension as a risk factor for CVD and its importance as a cause of premature mortality in South Africa, few studies to date have assessed the prevalence of hypertension and its risk factors in the rural areas of the country. Therefore the aim of this study was to determine the prevalence of and associated risk factors for hypertension amongst adults in the Dikgale Health and Demographic Surveillance Site (HDSS), Limpopo Province, South Africa

## Research methods and design

### Study design and population

A cross-sectional community-based study was carried out in the Dikgale HDSS, Limpopo Province, South Africa. The area consists of 15 villages, 7200 householdsand a population of approximately 36 000. Unemployment within the HDSS population is high, despite high rates of literacy and education. A detailed description of the study site has been provided elsewhere.^7,19,20^

### Inclusion and exclusion criteria

All individuals aged 15 years and older who permanently resided in Dikgale HDSS and had the ability to comprehend the contents of the interview were eligible to participate. Individuals who were non-cooperative or refused to provide the necessary information were excluded from the study. Pregnant women and bedridden and disabled individuals were also excluded. Members of families who were not present at the time of the assessment were excluded after three repeated visits.

### Sample size and sampling technique

A minimum sample size of 1366 (approximated to 1400) was calculated based on the reported hypertension prevalence rate of 46% in South Africa,^21^ a sampling error of 4%, 95% confidence, non-response rate (10%) and design effect set at 2. Each village was taken as a cluster, and all households within a cluster were visited. In a household all family members available at the time of data collection and eligible to participate were included in the study. A total of 1407 individuals gave their consent and participated in the study.

### Data collection

Data collection was carried out between June 2011 and March 2012 by trained field workers. The questionnaire used for the study was a modified World Health Organization STEPwise instrument for NCD risk factor surveillance.^22^ Blood pressure (BP) was measured three times using the Omron M6 Digital Automatic Blood Pressure Monitor (Kyoto, Japan), and the average of the three readings used for analysis. Height and weight were measured once using a stadiometer to the nearest 0.5 cm and the digital balance to the nearest 0.1 kg respectively. Participants were measured without shoes and wearing only light clothing. Body mass index (BMI) was calculated as weight in kg divided by height in metres squared (m²). BMI was then categorised as underweight (< 18.5 kg/m^2^), normal (18.5 kg/m^2^ – 24.9 kg/m^2^), overweight (25.0 kg/m^2^ –29.9 kg/m^2^) or obese (≥ 30 kg/m^2^).

Hypertension was diagnosed when systolic BP was ≥ 140 mmHg and/or diastolic BP ≥ 90 mmHg or when a person had a history of antihypertensive treatment during the survey. Isolated diastolic hypertension was defined as diastolic BP more than 90 mmHg and systolic BP less than 140 mmHg, whilst isolated systolic hypertension referred to systolic BP of more than 140 mmHg and diastolic BP of less than 90 mmHg.^23^

### Data analysis

Data entry and statistical analysis were performed using Microsoft Excel and statistical software (Stata 9.0, StataCorp, College Station, Texas, United States of America [USA]), respectively. Differences in demographic characteristics between hypertensive and normotensive groups were analysed using the Chi-square test. The student *t*-test was also used to compare the age distribution of males and females. A *p*-value of less than 5% was considered statistically significant.

## Ethical considerations

Ethics approval (Ref: MREC/HS/05/2013) to conduct the study was obtained from the University of Limpopo Ethics Committee (Medunsa Campus), and the anonymity and confidentiality of individual participants and personal information was protected.

## Results

### Demographic characteristics

A total of 1407 individuals were interviewed during the period of the study, of whom 1281 had complete information, giving a response rate of 91%. Nine per cent (126/1407) of the participants were excluded from the study because incomplete measurements of BP, weight or height were taken. The ages of participants ranged from 15 to 98 years, with a mean of 44.2 ± 20.9 years. Of the sample 42% was aged 50 years and older and 63% was female, as indicated in Table 1. The mean age of females was significantly higher than that of males (45.9 ± 20.2 vs. 41.3 ± 21.5, *p* < 0.001). Fifty-five per cent of the participants were single and 90% were unemployed, whilst 87% were non-smokers and 80% were non-alcohol drinkers (see Table 1).

**TABLE 1:** Demographic characteristics of the respondents (*n* = 1281).

Characteristic	Number	%
**Age (yrs)**		
15–19	159	12
20–29	294	23
30–39	132	10
40–49	144	11
50–59	182	14
60+	366	28
**Gender**		
Male	480	37
Female	801	63
**Marital status**		
Single	695	55
Married	456	36
Widowed	84	7
Others	34	3

### Prevalence of hypertension

The overall prevalence of hypertension was 41.4% (95% CI: 38.7% – 44.1%) with 21% (95% CI: 17.4% – 24.5%) having isolated systolic hypertension and 26% (95% CI: 22.2% – 29.8%) isolated diastolic hypertension. As displayed in Table 2, the prevalence of hypertension varied according to age group, marital status and level of education. As age increased, the prevalence of hypertension also increased (*p* < 0.05). Participants with tertiary education showed a significantly lower prevalence of hypertension compared to the other groups (*p* < 0.05). The prevalence of hypertension was found to be lower amongst single (35%) respondents compared to the other groups, the difference being statistically significant (*p* < 0.05).

**TABLE 2:** Association of sociodemographics with hypertension amongst respondents in Dikgale Health and Demographic Surveillance Site

Characteristic	*n*	Normotensive *N*(%)	Hypertension *N*(%)	*p*-value
**Age (yrs)**				
15–19	159	105 (66)	54 (34)	< 0.001
20–29	294	202 (68)	92 (31)		
30–39	132	81 (61)	51 (39)		
40–49	144	85 (59)	59 (41)		
50–59	182	94 (52)	88 (48)		
60 +	366	181 (49)	185 (51)		
**Gender**				
Female	801	473 (59)	328 (41)	0.690
Male	480	278 (58)	202 (42)		
**Employment status**				
Unemployed	1124	651 (58)	473 (42)	0.058
Employed	126	84 (67)	42 (33)		
**Marital status**				
Single	699	455 (65)	244 (35)	< 0.001
Married	456	230 (50)	226 (49)		
Widowed	84	44 (52)	40 (48)		
Divorced/separated	30	16 (53)	14 (47)		
**Level of education**				
None	148	72 (49)	76 (51)	< 0.001
Primary	600	328 (55)	272 (45)		
Secondary	487	317 (65)	170 (35)		
Tertiary	40	30 (75)	10 (25)		
**Alcohol**				
No	1025	600 (58)	425 (42)	0.812
Yes	251	149 (59)	102 (41)		
Smoking				
No	1112	647 (58)	465 (41)	0.516
Yes	166	101 (61)	65 (39)		
**Fruit and vegetable intake**				
< 5 servings/day	1139	666 (59)	473 (41)	0.752
> 5 servings/day	142	85 (60)	57 (40)		
**BMI (kg/m^2^)**				
< 18.5	68	45 (66)	23 (34)	0.310
18.5–24.9	544	325 (60)	219 (40)		
25.0–29.9	340	190 (56)	150 (44)		
≥ 30	294	165 (56)	129 (44)		

The proportion of participants with hypertension was found to be higher amongst males than females (42% vs. 41%, *p* > 0.05). An insignificantly greater proportion of unemployed respondents was hypertensive compared to employed respondents (42% vs. 33%, *p* > 0.05). Other factors, such as alcohol intake (*p* = 0.812), smoking (*p* = 0.516), fruit and vegetable intake (*p* = 0.752) and BMI (*p* = 0.310), were not significantly associated with hypertension (see Table 2).

## Discussion

In our study the overall prevalence of hypertension was 41%. In recent African studies the prevalence of hypertension in sub-Saharan Africa ranges between 5% and 50%.^11,12,24^ Our findings are similar to those reported in rural Ghana,^25^ rural Nigeria^26^ and the Agincourt rural subdistrict in SouthAfrica.^27^ However, the prevalence of hypertension in our study was higher than that in rural Ethiopia^28^ and rural Uganda.^29,30^ Our findings therefore confirm the growing concern about hypertension as a public health problem in rural South Africa.

Studies in developed countries revealed a large variation in the prevalence of hypertension, with a rate of 19.5% in Canada, 29% in the USA and 30% in England.^13^ In South Africa the national prevalence of hypertension has increased from 21% in 1998^14^ to 77.3% in 2008.^17^ This might be due to rapid urbanisation, ageing of the population, an increase in psychosocial stress, diet and lifestyle changes.^31^ There is evidence that the prevalence of hypertension in low- and middle-income countries is greater than in developed nations.^13^ This has negative implications since in many developing countries priority is given to acute disorders and maternal and child health. In addition, the health system is inadequate because of low funds, poor infrastructure and lack of equipment.

With regard to sociodemographics, studies reported that being overweight or obese, falling within an older age group, use of alcohol, being married and level of education are associated with hypertension.^25,26,29,30,31,32,33,34^ The findings of our study confirm that being married, low education level and falling within an older age group are the most common risk factors for hypertension. High BP is a multifactorial disorder, and changes with age might be due to changes in the vascular system,^35^ whilst poor partner support and marital conflict were shown to increase cardiovascular reaction and BP.^36,37,38,39,40^

Earlier studies suggest that a high BMI contributes to hypertension.^41,42,43,44^ Our study showed that the association between BMI and hypertension was not significant, which may partly be attributed to the declining use of dietary salt, despite the increase in obesity.^45,46^

In this study the prevalence of hypertension was slightly higher in men than in women, but the difference was not statistically significant. This finding is similar to those reported in other studies,^11,27,33^ but differed from those which found the prevalence of hypertension to be higher in women than men.^7,14,29,30,47^ Hypertension seems to coexist with the risk factors mentioned above, and thus may be an important clinical entity which requires medical attention. In the elderly it is well known that hypertension increases with vascular resistance. Therefore the therapeutic approach to hypertensive patients consists of a reduction in salt and caloric intake and an increase in physical activity.^48,49,51^

### Limitations

The following limitations need to be considered. As the scope of this study did not include biochemical measurements, we were unable to determine the levels of blood glucose or cholesterol, which were not assessed because the participants had not fasted. Finally, as this was a cross-sectional study no causal relationship can be inferred between any of the factors and hypertension.

## Conclusion

The prevalence of hypertension is relatively high in this rural community and is associated with being married, having a low education level and falling into an older age group. Given the importance of BP control in reducing the risk of CVD, there is an urgent need for strategies to promote BP screening at local health facilities as well as at community level and to promote prompt treatment initiation and follow-up of cases. In addition, salt reduction in food requires commitment at both provincial and national level.
